# Mallein as an Aid to the Diagnosis of Glanders

**Published:** 1893-02

**Authors:** W. Hunting, J. M’Fadyean

**Affiliations:** London; Royal Veterinary College, London


### MALLEIN AS AN AID TO THE DIAGNOSIS OF GLANDERS.

By W. Hunting, F.R.C.V.S., London, and

J. M'Fadyean, M.B., B.Sc., Royal Veterinary College, London.

Mallein is a liquid obtained by extracting cultures of the glanders bacillus with glycerine. Its preparation was suggested to the Russian veterinary surgeon Kalning by the analogous solution first introduced by Koch under the name tuberculin, the latter being a glycerine extract obtained from cultures of the bacillus tuberculosis. Kalning's experiments showed that, just as tuberculin when subcutaneously injected is capable of exciting a febrile reaction in tuberculous patients, so mallein determines a rise of temperature and other symptoms of fever in horses that are the subject of glanders. Moreover, as with tuberculin, this effect is specific, the mallein being without appreciable effect on healthy or non-glandered horses.

Kalning's observations soon attracted attention, and during the past eighteen months experiments with mallein on a very large scale have been carried out in Germany, France, and elsewhere. The results have been so uniformly favorable as to leave little doubt regarding its high diagnostic value.

The matter may thus be said to have passed the experimental stage, and, as there is probably no place in the world where glanders is more prevalent at the present time than in London, it appeared highly desirable to bring the value of mallein home to veterinary surgeons here. That is our object in publishing the following observations, for it is probable that even a small number of experiments made at our own doors are more likely to attract attention than the summary of a much larger series of cases from foreign literature.

For the mallein used in the following cases we are indebted to Dr. Roux, of the Pasteur Institute, Paris, and we desire to express here our gratitude for the promptness and liberality with which he acceded to our request to be supplied with a quantity of the material. We are also under obligation to fellow members of the profession in London who have aided us by placing at our disposal horses under their charge suspected of glanders. *

Mallein is sent out from the Pasteur Institute in two forms. The first of these, or diluted mallein, is a transparent straw-colored liquid, despatched ready for use in Pasteur tubes. This diluted mallein retains its activity for about a fortnight if the tubes are kept in the dark and unopened. The second form is the concentrated mallein; this is a thicker, deeper-colored liquid, and it retains its activity for some months. Before use it must be reduced to the condition of the diluted mallein by adding to it nine times its volume of a ½ per cent. solution of carbolic acid in water. The prescribed dose of diluted mallein for a horse is 2½cc., and the best seat for the injection is the middle of the side of the neck.

It is advisable to ascertain the temperature of the animal once or twice during the day preceding the inoculation, and in every case it must be noted at the time of inoculation, and several times during the course of the next 18 hours. Were it not for the trouble involved, one would recommend that the temperature should be taken every two hours during the 24 after inoculation, but in every-day-practice many would doubtless find this very irksome or even impossible. Fortunately a smaller number of observations may be made without much risk of missing any rise of temperature, for experience has shown that the febrile reaction seldom sets in during the first 2 or 3 hours, and that when once the temperature has become febrile it remains so for several hours. The practitioner may, therefore, content himself with taking the temperature once on the day prior to inoculation, at the hour of inoculation, and then at the 6th, 10th, 14th and 18th hours afterwards. At each observation after inoculation not only the temperature but also the local reaction (at the seat of inoculation) and any evidence of general disturbance should be noted.

Case I.—Donkey, aged; apparently healthy; dissection subject. At 12 o'clock, midnight, his temperature was 98.5; he had

* Mr. Hobday, M.R.C.V.S., and Messrs. Hunting and Calestreme, veterinary students, rendered valuable assistance in the taking of temperatures.

received 2 cc. mallein. Six hours later his temperature remained the same; a firm swelling at seat of inoculation, about the size of a walnut was noticed. At 3 p. m., his temperature had risen to 100, and his appetite was good, and general appearance normal. The next morning at 9 o'clock, his temperature had fallen to 98.5, and the local swelling was scarcely perceptible.

A few days later the donkey was killed, and its subsequent dissection showed it to be free from glanders lesions.

Case II.—Chestnut pony, aged; very emaciated, but apparently healthy; purchased for dissection. At 11:45 p. m., his temp. was 99.8; he had received 2 cc. mallein. Six hours later his temp. having fallen to 98.5. a swelling at seat of inoculation about the size of a walnut was noticed. which was very tender to the touch. Twelve hours later his temp. had risen to 100.8; his good appetite was maintained, but swelling continued about the same. The next morning at 9 o'clock his temp. was 100, and the swelling was smaller, but very sensitive. Killed for dissection.

No glanders lesions were discovered during the dissection of this subject.

Case III.—Bay cart gelding, aged; submaxillary glands tumefied. Slight erosion in left nostril; bad grunter. General appearance unthrifty. Off hind leg thickened; has a number of discharging buds on inside of limb between hock and stifle. At 12 o'clock, noon, his temp. being 100, he had received 2½ cc. mallein. After six hours his temp. had risen to 102.8; pulse intermittent; feeding well; swelling at seat of inoculation 6 in. x 3 in. Four hours later the temp. had risen to 103.5, and swelling was tender to pressure. At 6 a. m., the next morning, the temp. had fallen to 103, and swelling extended downwards over jugular furrow. At 10 a. m., the temp. being the same, it was noticed that he left part of last feed; pulse very intermittent; swelling tender and somewhat fluctuating.

The swelling at seat of inoculation gradually decreased. and no noteworthy alteration in the animal's general condition took place until the ninth day after observation. At about 11 p. m., on that date, he was noticed to be in very acute pain, sweating profusely, and gasping for breath. Death took place the following morning at 3 a. m.

*Post-mortem* revealed glanders nodules in the lungs. The septum nasi was not examined.

Case IV.—Roan cart gelding; apparently healthy. At 12 o'clock, noon, his temp. was 100.4; he had received 2½ cc. mallein.

Six hours later his temp. had risen to 100.8, and appetite was noted good; swollen at seat of inoculation. The next morning at 10:30 the swelling disappeared, and temp. was 100.4; feeding well.

This animal is still alive, and apparently healthy.

CASE V.—Chestnut cart gelding; discharge from both nostrils. Tumefaction of submaxillary lymphatic glands on both sides; glanders ulcer visible in left nostril. At 12 o'clock, noon, temp. was 102.2, having received 2½ cc. mallein. Six hours after temp. had risen to 103.6; slightly off feed. At 10.30 p. m., temp. remaining the same; a swelling at seat of inoculation was noticed. The next morning at 10:30 the temp. was 103; not feeding well. Slaughtered.

*Post-mortem.*—Right lung contains numerous glanders nodules. Left lung, no perceptible glanders nodules. Septum nasi on left side much eroded and ulcerated. Ulcer on right vocal cord.

CASE VI.—Black aged pony, purchased for dissection; apparently healthy, appetite good. At 2 p. m., temp. was 100. At 11 p. m., temp. was 106, having received 2½ cc. mallein. Next morning at 5 o'clock a flat swelling at seat of inoculation, 5 inches in diameter, which was intensely painful, was noticed, temp. being the same as at 11 p. m. At 2 o'clock that day temp. was 102.2, and swelling was decreasing; 5 p. m., swelling less sensitive and small; temp. 100.8. At 8 p. m., temp. 99.8; swelling painless. At 11 p. m., that night, temp. was 99.2. Slaughtered.

No glanders lesions were discovered in the course of dissection.

CASE VII. Chestnut cart mare; subject of nasal gleet. Admitted into the college infirmary. At 10 a. m., temp. was 101; nostrils were fumigated with Tuson's disinfectant and ol. tereb., aa. At midnight temp. was 100.8; received 2½ cc. mallein. Six hours later a swelling at seat of inoculation about size of walnut was noted; feeding well; temp. the same. At noon that day temp. was 101.2, and swelling perceptibly less. The next morning at 9 o'clock the temp. was 100: swelling had quite disappeared; no nasal discharge. Discharged cured.

CASE VIII.—The history of the following case case is peculiarly interesting. The animal, a bay cart-gelding, was purchased to serve as a subject in the class of elementary clinique, in which junior students practise, among other things, the administration of balls. The horse was specially chosen as being one without any suspicion of glanders, but his history was not obtainable. After he had stood in the college for a day or two it was noticed that he had a slight discharge from the right nostril, and it was therefore determined to test him with mallein. At 9 p. m., temp. was 101.4; pulse very irregular and intermittent; received 2½ cc. mallein subcutaneously on near side of neck. At 3 a. m., temp. was 101; swelling at seat of inoculation about 3 inches in diameter; not very sensitive. At 8 o'clock that morning temp. was 101.8; appetite good; aspect lively; pulse very intermittent; swelling about same size, but more sensitive. By 3 o'clock that afternoon swelling had become very painful, and temp. was 105. At 9 p. m., temp. had fallen to 104.6; breathing labored. At midnight temp. was 104.4; breathing still labored; horse standing constantly in one position; pulse very intermittent. At 10:30 the next night the temp. was 103; swelling more diffused and very painful. Next morning at 9 o'clock temp. was 102; feeding well; whitish discharge from both nostrils. Next day, 9 a. m., temp. 99.5; animal down and unable to get up; appetite retained. Slaughtered.

*Post-mortem.*—Numerous glanders nodules varying in size from a hazel nut to a walnut in both lungs. One recent nodule in septum.

Case IX.—Roan cart gelding, 8 years old. Near hind leg thickened, with discharging farcy bud on inside of hock. Appetite good. At 3.30 p. m. temp. was 102.4; next morning at 11 temp. was 101.4; having received 2½ cc. mallein; 5 p. m., temp. 103.8; rigors; seat of inoculation swollen; off feed; 11 p. m., temp. 105.8; seat of inoculation intensely painful; swelling 3 or 4 inches in diameter. Two days after, temp. 105; appetite better; swelling slightly decreased. Next day at 10 a. m., temp. 103.2; swelling remaining about same size; intensely painful. Two days after this temp. was 101.6; appetite suppressed; near fore leg swollen; brachial lymphatic glands appear enlarged.

Three weeks later this animal was injected for the second time. At this date the following notes were made regarding his condition:

Appetite good. On both inner and outer aspects of near foreleg in the region of the knee, there are numerous farcy sores. Near hind hock shows an old healing ulcer, and behind that several recent sores. On the back of the hock there is a quite recent bud. Animal grunts badly when threatened.

Second Injection.—At 12 o'clock, noon, temp. 100 5; injected with 2½ cc. mallein. Six hours later, temp. 103; pulse very weak; animal off feed; swelling at seat of inoculation well defined, 4 in. x 3 in.; very tender. At 10 p. m., temp. 105.1; swelling larger, pits on strong pressure. At 2 a. m. next day, temp. 104.4; has eaten part of a feed. At 10 a. m., temp. 103.2 ; animal off feed ; swelling very painful.

This animal was killed a week after the last observation.

*Post-mortem.*—Lungs contained a few small glanders nodules. Typical farcy buds under skin of knee of near fore limb and hock of near hind limb. Several typical glanders ulcers on left side of septum nasi ; these evidently of very recent formation.

Case X.—Aged chestnut pony purchased for dissection. Very emaciated, but apparent healthy. At 11.45 p. m., temp. 100.6 ; received 2½ cc. mallein. Six hours later, temp, 99.8 ; firm swelling size of walnut at seat of inoculation. Three hours later, temp. 99; eating well Three o'clock that afternoon, temp. 101; appetite good. Three hours later, temp. 102.5; appetite good ; swelling as before. Next morning 9 a. m., temp. 101.4; swelling diminished in size but more painful; 4 p. m., temp., 101.6; swelling almost disappeared. Next day, 10 a. m., temp. 102.4; feeding well.

This animal was killed a few days later, and no glanders lesions were discovered during its dissection.

Case XI.—Black pony mare brought to free clinique. Detained on account of a discharge from the right nostril. Submaxillary gland of same side as large as a walnut and adherent to the jaw. Did not grunt when threatened 6 p. m., temp. 103.1; received 2½ cc. mallein; midnight temp. 102.8; flat swelling at seat of inoculation, 3 in. x 2 in.; 4 a. m., temp. 102.6, appetite good; 8 a. m., temp. 102 8, appetite maintained; swelling smaller, not painful.

The pony was slaughtered at 11 a. m. on the 22nd November.

*Post-mortem* —A large erosion high up on the septum nasi. The lungs contained numerous glanders nodules.

Since in this case there was no general reaction and only an unimportant local one, it appeared very desirable to obtain positive evidence regarding the nature of the lung lesions, although their appearance left little room for doubt that they were glanderous. A guinea-pig was therefore subcutaneously inoculated on the abdomen with matter from one of the lung nodules, and it died 24 days afterwards with glanders lesions in the left testicle, left inguinal lymphatic gland, and nose. Pure cultures of the glanders bacillus were obtained from the lymphatic gland.

Case XII.—Brown pony, aged, brought to free clinique. The animal was detained on account of suspicious unhealthy-looking sores on the external skin of the left nostril. Both submaxillary glands were enlarged to the size of a walnut and adherent to the jaw. No visible lesions in nose. Midnight temp. 99.6; received 2½ cc mallein; 6 a. m , temp. 103; firm swelling, 3 in. x 2 in., at seat of inoculation; 1 p. m., temp. 105; not feeding; very dull; head held down; swelling very tense and painful; 5 p. m., temp. 104 8; swelling somewhat increased in size. Pony slaughtered.

*Post-mortem.*—Septum nasi shows on left side 8 or 9 recent glanders ulcers; on right side some small ecchymoses, but no ulcers. Right lung contains numerous glanders nodules, and its pleural covering shows several patches of thickening. Left lung contains some nodules of cartilaginous consistence.

Case XIII.—Chestnut mare, aged, brought to the free clinique. Detained as suspected of glanders. The animal appeared very weak and ill. There was a white flaky discharge from the right nostril, and a similar discharge from the inner canthus of right eye. The left mammary gland was swollen and firm, about as large as the closed fist ; a few inches in front of this, near the umbilicus, there was a swelling of about the same size but more oblong in shape. In the backward direction this was continuous by means of a subcutaneous cord with the enlarged mammary gland. At 10 a. m., temp. 103.4. Next day, 4 p. m., temp. 103.0 ; received 2½ cc. mallein. Midnight, temp., 102.6 ; swelling at seat of inoculation about size of walnut. 4 a m., temp. 100 ; animal down and unable to get up; 9 a. m., temp. 100 ; animal in extremis. Slaughtered.

*Post Mortem.* —Mammary gland contains numerous small purulent centres. The swelling in region of umbilicus proved to be due to inflammatory thickening of the subcutaneous tissue, without any suppuration. Recent pleurisy with adhesion of left lung to chest wall by means of a creamy fibrinous layer. Both lungs crammed with glanders nodules.

Case XIV.—This is in some respects the most interesting and instructive case in the series. After the horse purchased for use in the class of elementary clinique had unexpectedly proved to be glandered (*see* Case VIII), it became apparent that great care would have to be exercised in providing for this purpose any animal whose previous history could not be ascertained. After some trouble there was obtained a mare whose history appeared to give a guarantee that she was free from glanders. This animal was an aged bay draught mare, which had been in possession of its last owner for seven years. At no part of that time had the mare shown the least suspicion of glanders or farcy, but she had become broken-winded, and for that reason was placed on the cast list.

There was no nasal discharge, the submaxillary glands were normal, and the skin was clean, but the general appearance somewhat unthrifty. The respiratory movements were characteristic of broken wind, and she grunted badly when threatened with a stick. She was a rather poor feeder. Prior to the injection of mallein it was observed that the pulse was frequent and weak, and that the temperature was abnormally high. Notwithstanding these symptoms, no suspicion of glanders was entertained. 6 p. m., temp. 103; received 2½ cc. mallein; 12 midnight, temp. 102 8; swelling at seat of inoculation, 3 in. x 4 in., slightly sensitive; 4 a. m. next day, temp. 104.4, feeding well; 8 a. m., temp. 104 4, swelling nearly gone, not feeding; 5 p. m., temp. 104.6, appetite fair; 11 p. m., not feeding well, swelling painful; a second swelling (prescapular lymphatic glands) about size hen's egg; next day, 9 a. m., temp. 104; swelling at seat of inoculation larger, 6 in. x 4 in., flat and not well defined; coat staring, and general appearance very unthrifty; 10 p. m., temp. 103.4, swelling still larger, pits on pressure.

From this time onwards the temperature fluctuated between 101.2° and 104.0°. The respirations remained as before, but the pulse was scarcely so frequent. Two days after the last observation an abscess the size of a mandarin orange appeared high up on the side of the left thigh. This was lanced, and microscopic examination of the pus after staining with methyl-blue, revealed a few bacilli resembling those of glanders. Cultures from this pus were made on sterilized potato and placed in the incubator. After three or four days an amber yellow growth made its appearance, and this subsequently underwent the deepening of colour characteristic of cultures of the bacillus mallei. The swellings in the neck gradually disappeared, but left the skin somewhat thickened. Subsequently the near hind leg became swollen ; the abscess on the inner aspect of the thigh continued to discharge, and farcy buds discharging a dirty yellowish purulent fluid made their appearance above and below the hock on the inside of the same limb. The appetite became very poor, the general appearance of the animal very unhealthy, and a fluctuating swelling appeared underneath the sternum. At no time was there the least nasal discharge, nor were the submaxillary lymphatic glands in any way affected. The mare was slaughtered on the 17th day after the inoculation.

*Post-mortem.*—Both lungs crammed with characteristic glanders nodules, many as large as a hazel nut. Considerable emphysema towards the apices and edges of the lungs. Liver cirrhotic and showing an early stage of nutmeg lesion. Spleen swollen, pulp firm, no visible lesions. No glanders lesions in nostrils. Farcy lesions (see above) on near hind leg.

CASE XV.—Grey gelding, aged. Suspected for three months; been in contact with diseased animals. Stellate scar on nasal membrane. 11 a m.. temp. 100.3 ; received 2½ cc. mallein ; 10 p. m., temp. 104.5, off feed and blowing ; 2 a. m., temp. 104, swelling at seat of injection ; 11 a m., temp. 103 6 ; very lame, off feed, neck swollen.

CASE XVI.—Grey gelding, aged. Suspicious for some weeks; maxillary gland enlarged; has had slight intermittent nasal dis charge. 11 a. m., temp. 99.2; received 2½ cc. mallein; 10 p. m. temp. 105.8, off feed and very dull; 2 a. m., next day, temp. 105.4. Inoculation spot swollen; 11 a. m., temp. 105.2; lame, excessive staling.

Remains alive; no visible signs of disease. Excessive action of kidneys lasted for a week.

CASE XVII.—Roan gelding, aged. Been under suspicion for 14 days on account of slight nasal discharge, which had now ceased. Maxillary glands slightly enlarged. 11 a. m., temp. 100 8; received 2½ cc. mallein ; 6 p. m., 103.2, off feed and dull; 10 p. m., blowing and discharge from nose; next morning at 11 o'clock, temp. 100. Inoculation spot swollen; very lame.

*5th Nov.*—Slaughtered. *Post-mortem*—Chronic glandered lungs; extensive ulceration of septum nasi.

CASE XVIII.—Brown mare, aged. Under suspicion for nasal discharge which ceased four months ago; also thickened off fore leg, but no "cords" or "buds." 11 a. m., temp. 100.8, received 2½ cc. mallein; 10 p. m., temp. 105; dull and off feed; next day, 11 a. m., temp. 104.2, feeding well, pulse 44.

Remains alive; inoculation spot never was swollen.

CASE XIX.—Grey mare, aged. Been suspected for some weeks; thickened off hind leg; no other sign of disease. 11 a. m., temp. 101.8; received 2½ cc. mallein; 10 p. m., temp. 105.4; blowing and very dull; next day, 2 a. m., temp. 105.6; off feed; inoculation spot swollen; 11 a. m., temp. 105.5; off hind leg much swollen; pulse 60.

*9th Dec.*—Slaughtered. No nasal disease; chronic lung lesions of glanders.

CASE XX.—Grey gelding, aged. Under suspicion. Has had occasional intermittent nasal discharge; no other sign of disease. 11 a. m., temp. 99.2 ; received 2½ cc. mallein ; 6 p. m., temp. 104.3; off feed and dull; 10 p. m., temp. 105; dull and blowing.

Next day at 2 a. m., temp. 104.2; inoculation spot much swollen; still blowing; 11 a. m., temp. 104; excessive staling; pulse 62.

Remains alive; no sign of disease.

CASE XXI.—Brown mare, aged. No sign of disease; lame from picked-up nail. Had enlarged gland and nasal discharge six years previously. Was then considered infected and ever since been watched as suspicious. 11 a. m., temp. 100.8; received 2½ cc. mallein; 6 p. m., temp. 102.4; off feed and dull; 10 p. m., temp. 103.6; blowing; 2 a. m., temp. 104.6; dull excessive staling; 11 a. m., temp. 103.6; great depression, continued staling.

Remains alive. Loss of appetite and great loss of condition were noticeable for a fortnight. No sign of disease developed; resumed work.

CASE XXII.—Chestnut mare, aged 6 years. Suspicious sore on off shoulder; been doing badly for some few weeks. 11 a. m., temp. 101; received 2½ cc. mallein; 10 p. m., temp. 105; off feed and blowing; 2 a. m., temp. 104.6; inoculation spot swollen; 11 a. m, temp. 104; dull, stiff and much swollen.

The constitutional disturbance remained marked for some days.

*6th Dec.* Slaughtered. Wound on shoulder, which had been cicatriced, again ulcerated and corded. Lungs studed with glanderous nodules.

CASE XXIII.—Brown gelding, 5 years. Enlarged maxillary gland and nasal discharge; been working with a known glandered horse. 2 p. m., temp. 102.8; received 2½ cc. mallein; 12 m., temp. 106; off feed; 6 a. m., temp. 105; feeding slightly; 11.30 a. m., temp. 104; not much disturbance; inoculation spot considerably swollen.

*7th Nov.*—Slaughtered. Ulcerated nasal membrane, and glanderous nodules in both lungs.

CASE XXIV.—Brown mare, aged. Slightly enlarged gland; poor in condition and slight nasal discharge; isolated as very suspicious. 2 p. m., temp. 102; received 2½ cc. mallein; next day at 11.30 a m, temp. 101.8; no constitutional disturbance and never off feed or blowing.

*12th Nov.*—Slaughtered. Ulcerated nasal membrane; glanderous nodules in both lungs, some caseous, others calcareous.

CASE XXV.—Brown mare, aged. Apparently healthy. 12 noon, temp. 99.4; received 2½ cc. mallein; temp. rose to 103.6, but remained lively, feeding and undisturbed; no swelling at seat of inoculation.

Remains alive and at work.

Case XXVI.—Chestnut mare, aged. Apparently healthy. 12 noon, temp 100.2; received 2½ cc. mallein; temp. fluctuated, going as high as 102.8, but remained lively, continued feeding inoculation spot not swollen.

Remains alive and at work.

Case XXVII.—Black gelding. Apparently healthy; wearing a tracheotomy tube. 12 noon, temp. 100.4; received 2½ cc. mallein; temp. fluctuated from 101.6 to 100; remained lively, continued feeding ; inoculated spot not swollen.

Remains alive and healthy.

Case XXVIII.—Bay gelding, aged. Apparently healthy, except for a thickening of hind leg resulting from accident. Received 2½ cc. mallein; 12 o'clock noon, temp. 100; fluctuated from 100 to 101.2; remained lively, continued feeding; inoculated spot not swollen.

Remains alive and healthy.

Case XXIX.—Bay gelding, aged. Looked unthrifty; had been turned out to grass for being weak and out of condition. 12 p. m., temp. 101.5; received 2½ cc. mallein; 6 p. m., temp. 105.6, dull and blowing; 10 p. m., temp. 105.2, inoculation spot swollen; 2 a. m., temp. 104.6; remained dull and disturbed ; 11 a. m., temp. 104; remained dull and disturbed and excessive staling.

*9th Nov.*—Slaughtered. Enlarged nutmeg liver; no glanderous changes in lung; nasal membrane congested but not ulcerated.

Case XXX.—Black mare, aged. Poor and unthrifty; no definite sign of disease. 1 p. m., temp. 101.2; received 2½ cc. mallein; 11 p. m., temp. 104 8, off feed, blowing and dull; 3 a. m., temp. 104.6, dull, and blowing; 11 a. m., temp. 104; inoculation spot much swollen.

*7th Nov.*—Slaughtered. Chronic glanderous lesions in lungs.

Case XXXI.—Black gelding, aged. Slightly enlarged maxillary gland; no other sign of disease. 1 p. m., temp. 99.8; received 2½ cc. mallein; temp. fluctuated 99.8 to 104.8; no swelling at inoculation spot and very slight constitutional disturbance.

*7th Nov.*—Slaughtered. Lung changes due to a previous pneumonic attack; no definite glanders lesions detected.

Case XXXII.—Chestnut gelding, aged. Apparently healthy. 1 p. m., temp. 100.2; received 2½ cc. mallein; temp. fluctuated from 100 to 101.2; remained lively, continued feeding, inoculated spot not swollen.

Remains alive and healthy.

Case XXXIII.—Black mare, 5 years old. Apparently healthy.

1 p. m., temp, 100.2; received 2½ cc. mallein; temp. fluctuated from 100.2 to 101.2; remained lively, continued feeding, inoculated spot not swollen.

Remains alive and healthy.

CASE XXXIV.—Chestnut gelding, aged. Fœtid nasal discharge; slightly enlarged glands. Diagnosed as a local disease. 11 a. m., temp. 100.2; received 2½ cc. mallein; temp. fluctuated from 100.2 to 102.3; remained lively, continued feeding, inoculated spot not swollen.

Remains alive; symptoms unaltered.

CASE XXXV.—Bay gelding, aged 6 years. Swollen off hind leg. Farcy buds and "corded" swelling on near side of neck. 11 a. m., temp. 102; received 2½ cc. mallein; 7 p. m., temp. 104.6; dull, blowing and off feed; 6 a. m., next day, temp. 105; hind leg much swollen, and the inoculation spot swollen also.

*12th Dec.*—Slaughtered. Ulceration of nasal membrane, and glanderous nodules in lung.

CASE XXXVI.—Brown gelding. Suspected on account of chronic cough and general unthrifty appearance. Had two attacks of pneumonia during the last six months. 11.30 a. m., temp. 101.2; received 2½ cc. mallein; temp. remained same for next fifteen hours; no evidence of general disturbance at any time, and very little swelling at seat of injection.

This horse is still alive, and his condition has improved since he was treated with mallein.

CASE XXXVII.—Bay gelding. About a month prior to inoculation farcy buds had formed along the abdomen in front of the prepuce. The animal seemed stiff in his hind legs. 11.30 a. m. temp. 102; received 2½ cc. mallein; temp. fluctuated from 103 to 105 in the next fifteen hours, a large painful swelling formed at the seat of inoculation, and the animal fed badly.

*7th Dec.*—Slaughtered. Lung full of glanders nodules; liver cirrhotic.

CASE XXXVIII.—Brown gelding, with slight discharge from right nostril and slight enlargement of submaxillary glands. An accidental wound inflicted on chest-wall two months previously had taken on an unhealthy action, and had absolutely refused to heal. Of late the horse had been very unthrifty. 11.30 a. m., temp. 101; received 2½ cc mallein; at 7.30 p. m. temp. was 105.1; 11.30 p. m., temp. 106; seat of inoculation much swollen; 3.30 a. m., temp. 105.3.

*7th Dec.*—Slaughtered. Glanders nodules in lungs.

Case XXXIX.—Bay gelding. Object of suspicion from sore on hock. 11.30 a. m., temp. 101; received 2½ cc. mallein; in the next twelve hours temp. remained almost the same, it going however to 101.3 at 11.30 p. m.

This horse was a vicious kicker and it became impossible to take his temperature after the 4th observation. The sore on the hock has healed, and it has since been learned that it was due to the application of an irritant. A few days after the injection with mallein an abscess burst on the inner aspect of one thigh, and that has healed up.

It now remains to make a brief analysis of the results obtained in these 39 cases. Fifteen of the horses are still alive, and the remaining 24 have been killed and submitted to a *post-mortem* examination. In 18 of these 24 distinct glanderous lesions were discovered, and in the other 6 the *post-mortem* did not reveal any such lesions. The following table refers to the 18 glandered horses.

| *No. in Series.* | *Initial Temperature.* | *Maximum Temperature during the 18 hours after injection.* | *Hour at which Temp. reached its maximum.* |
|---|---|---|---|
| 3 | 100.0 | 103.5 | 10th |
| 5 | 102.2 | 103.6 | 6th |
| 8 | 101.4 | 105.0 | 18th |
| 9 | 101.4 | 105.8 | 12th |
| 11 | 103.1 | 102.8 | 14th |
| 12 | 99.6 | 105.0 | 13th |
| 13 | 103.0 | 102.6 | 8th |
| 14 | 103.0 | 105.0 | 18th |
| 15 | 100.3 | 104.5 | 11th |
| 17 | 100.8 | 105.2 | 11th |
| 19 | 101.8 | 105.6 | 14th |
| 22 | 101.0 | 105.0 | 11th |
| 23 | 102.8 | 106.0 | 10th |
| 24 | 102.0 | 103.0 | 10th |
| 30 | 101.2 | 104.8 | 10th |
| 35 | 102.0 | 105.0 | 13th |
| 37 | 102.0 | 105.0 | 8th |
| 38 | 101.0 | 106.0 | 12th |

A glance at the above table will show that except in two instances (Nos. XI and XIII) the temperature after the injection of mallein went to 103° at least; in three cases it reached 103-104°, in 2 cases 104-105°, and in 11 cases 105-106°.

In the two cases in which no rise of temperature followed, the latter was febrile (103°) before the injection of the mallein, and in one of these (No. XIII) the animal was almost in a dying state at the time of injection.

Turning next to the 6 cases in which the *post-mortem* did not reveal any glanderous lesions, it will be observed from the following figures that in 3 of them the temperature after injection remained under 103°, while in the other 3 it rose to that point or above it.

| *No. in Series.* | *Maximum Temperature.* |
|---|---|
| 1 | 101.0 |
| 2 | 100.8 |
| 6 | 103.0 |
| 10 | 102.5 |
| 29 | 105.6 |
| 31 | 104.8 |

Finally, there remain for notice the 15 cases in which the animals are still alive. The following table refers to them. The mark + denotes a clinical suspicion of glanders, — that the animal was believed to be healthy or non-glandered.

| *No. in Series.* | *History.* | *Initial Temperature.* | *Maximum Temperature after Injection.* |
|---|---|---|---|
| 4 | — | 100.4 | 100.8 |
| 7 | — | 100.8 | 101.8 |
| 16 | + | 99.2 | 105.8 |
| 18 | + | 100.8 | 105.4 |
| 20 | + | 99.2 | 105.0 |
| 21 | — | 100.8 | 104.6 |
| 25 | — | 99.4 | 103.6 |
| 26 | — | 100.2 | 102.8 |
| 27 | — | 100.4 | 101.6 |
| 28 | — | 100.0 | 101.2 |
| 32 | — | 100.2 | 101.2 |
| 33 | — | 100.2 | 100.6 |
| 34 | — | 100.2 | 102.3 |
| 36 | — | 101.2 | 101.2 |
| 39 | — | 101.0 | 101.3 |

We do not intend to appraise the precise value of mallein from the results obtained in these experiments, but prefer to wait until we can base our estimate on a larger series of observations. The readers of this article will probably form their own conclusions on the matter, but in doing so two points should be kept in mind. The first is that the majority of the horses discovered to be glandered on *post-mortem* did not present during life any symptom from which the most experienced practitioner could have certainly diagnosed the disease. The second point is that the non-discovery of glanders lesions in certain of the cases is not absolute proof of their non-existence. In most of the cases the *post-mortem* examination had to be carried out at a knacker's, and it had sometimes to be of a rough-and-ready character.

